# How is inclusiveness in health systems research priority-setting affected when community organizations lead the process?

**DOI:** 10.1093/heapol/czac012

**Published:** 2022-02-16

**Authors:** Bridget Pratt, Prashanth N Srinivas, Tanya Seshadri

**Affiliations:** Queensland Bioethics Centre, Australian Catholic University, Brisbane, 1100 Nudgee Road, Banyo, QLD 4014, Australia; Centre for Health Equity, School of Population and Global Health, University of Melbourne, Melbourne, 207 Bouverie Street, Carlton, VIC 3053, Australia; Institute of Public Health, 3009, II-A Main, 17th Cross, Krishna Rajendra Road, Banashankari Stage II, Bengaluru, Karnataka 560070, India; Institute of Public Health, 3009, II-A Main, 17th Cross, Krishna Rajendra Road, Banashankari Stage II, Bengaluru, Karnataka 560070, India; Vivekananda Girijana Kalyana Kendra, BR Hills, Chamarajanagar District, Karnataka 571441, India

**Keywords:** Health research, priority-setting, inclusion, community organization, engagement, involvement, partnership, indigenous

## Abstract

Community engagement is gaining prominence in health research. But communities rarely have a say in the agendas or conduct of the very health research projects that aim to help them. One way thought to achieve greater inclusion for communities throughout health research projects, including during priority-setting, is for researchers to partner with community organizations (COs). This paper provides initial empirical evidence as to the complexities such partnerships bring to priority-setting practice. Case study research was undertaken on a three-stage CO-led priority-setting process for health systems research. The CO was the Zilla Budakattu Girijana Abhivrudhhi Sangha, a district-level community development organization representing the Soliga people in Karnataka, India. Data on the priority-setting process were collected in 2018 and 2019 through in-depth interviews with researchers, Sangha leaders and field investigators from the Soliga community who collected data as part of the priority-setting process. Direct observation and document collection were also performed, and data from all three sources were thematically analysed. The case study demonstrates that, when COs lead health research priority-setting, their strengths and weaknesses in terms of representation and voice will affect inclusion at each stage of the priority-setting process. CO strengths can deepen inclusion by the CO and its wider community. CO weaknesses can create limitations for inclusion if not mitigated, exacerbating or reinforcing the very hierarchies that impede the achievement of improved health outcomes, e.g. exclusion of women in decision-making processes related to their health. Based on these findings, recommendations are made to support the achievement of inclusive CO-led health research priority-setting processes.

**Key messages**

- Partnerships between researchers and community organizations are thought to deepen communities’ inclusion throughout health research projects, including during priority-setting.
- This case study demonstrates that, when community organizations lead health systems research priority-setting, their strengths and weaknesses in terms of representation (range and mass) and voice (having a say and being heard) will affect community inclusion at each stage of the priority-setting process.
- Recommendations are made to support the achievement of inclusive community organization–led health research priority-setting processes.

## Introduction

Community engagement is gaining prominence, with funders, particularly those of applied health research, and research institutions increasingly expecting researchers to engage communities throughout the research process (Fleurence *et al.*, 2013; van Bekkum and Hilton 2014; Oswald *et al.*, 2016). This entails involving communities not only when shaping research projects’ design, conduct and dissemination but also when setting their research topics and formulating their research questions (Woolf *et al.*, 2016). Such engagement is seen as a key way to ensure that research projects ask the ‘right’ questions—those that are responsive to pressing community-identified needs—and create ‘better’ knowledge that draws on and reflects a diversity of knowledge systems and is more widely shared—beyond peer-reviewed journals and academic conferences (Hall *et al.*, 2011). It has ‘the potential to… compensate for or resolve existing differences in power, privilege, and positionality; [and] allow for marginalized voices and experiences to be represented in the production of scientific knowledge’ (Reynolds and Sariola 2018, p. 257).

Yet, communities rarely have a say in the agendas or conduct of the very health research projects that aim to help them. Typically, research projects’ agendas and design are defined by funders and researchers, often from high-income countries. One way thought to achieve greater inclusion for communities in research, including during priority-setting, is for researchers to partner with community organizations (COs). Accordingly, many universities now have engagement strategies that (amongst other things) call for undertaking research in partnership with COs. In the United States and Canada, a growing trend is to require such partnerships as a condition for research grants (Williams *et al.*, 2005; Fleurence *et al.*, 2013). COs often represent and/or have strong networks with the communities they serve, including those considered marginalized, which have been developed through grassroots work and outreach activities.

But is achieving inclusion simply a matter of partnering with a CO that is committed to improving its community? Does partnering with COs guarantee/promote greater inclusion of communities in health research priority-setting? Key dimensions of inclusion identified in the philosophy and development studies literature are representation (range and mass) and voice (having a say and being heard). Inclusion means achieving ‘range’: those present reflect the diversity of the community; all those affected by a decision are present, including those considered disadvantaged or marginalized (Young, 2000; Goulet, 2006; Goodin, 2008). Beyond who is present, David A. Crocker (2008) notes that the ‘mass’ or numbers of different types of participants are also important. The aim is to ensure that more powerful actors within a given community do not dominate decision-making by force of numbers. Iris Marion Young (2000) argues that deep inclusion is also determined by how those invited to be present for a decision-making process are involved: do they have an equal opportunity to share their ideas during the process? Andrea Cornwall (2011) further highlights a distinction between being able to raise one’s voice and being listened to and heard in decision-making processes. ‘Raising voice’ means being able to share one’s ideas and perspectives, while ‘being heard’ means having them reflected in the final output of a decision-making process.

This paper explores how power sharing within a CO affects inclusion (representation and voice) of its leaders and the wider community during a health systems research priority-setting process led by the CO. The choice of the case under study was based on a response to an open collaboration call to examine marginalized communities’ inclusion in research priority-setting. The case study research was pitched as a collaboration with those undertaking the projects that functioned as cases. PNS and TS responded to the open call and proposed the case under study. Case study research was then undertaken on a CO-led three-stage research priority-setting process to identify an indigenous population’s maternal and child health system priorities and interventions to address them. The priority-setting process was conducted in the Biligiriranga Hills in India. It is important to note that PNS and TS’s decision to critically analyse and reflect on this process was made after the priority-setting process had largely finished, due to the timing of the opportunity.

The partnership in the case under study was between researchers from Vivekananda Girijana Kalyana Kendra, a non-governmental organization (NGO) that has been working for the development of Soliga people since 1981; researchers from the Institute of Public Health, a research institute in Bangalore; and leaders of the Zilla Budakattu Girijana Abhivrudhhi Sangha, a district-level community development organization representing the Soliga people (see Box 1). The partnership began in 2014 through the project under study, with the researchers also making a long-term commitment to continue working with the Sangha beyond that project through a research field station located within the community.^1^ (In this paper, the community refers to the Soliga people (and other indigenous people) living in Biligiriranga Hills. Definitions of “community” can be based on geography; on special interests or goals; or on shared experiences, characteristics, or ethnicity (Molyneux and Bull 2013)). The researchers recognized that, while setting up a new platform could have potentially pushed the boundaries of engagement in terms of hierarchies and power dynamics within the community, such platforms often end up seeding new short-term engagement partnerships that primarily serve research organizations’ agendas. To avoid the latter, the researchers sought to critically engage with an existing CO: the Sangha. They also chose to work with the Sangha because it had demonstrated broad-based representativeness. The Sangha is several decades old and has a federated and representative organizational structure that goes down to every Soliga settlement in the Biligiriranga Hills. The Sangha’s commitment to improving the lives of the Soliga people and its leadership was evident in its decades-long but ultimately successful struggle to secure forest rights for the Soliga people (Tatpati and Pathak-Broome 2016). Furthermore, it had a track record of partnering with NGOs and academia in its work related to forest rights.

**Box 1.** The Soliga people

The Soliga are an indigenous population designated as a ‘Scheduled Tribe’ under the Indian Constitution’s statutory provisions to recognize vulnerable groups for affirmative action. The Soliga are among several indigenous communities in India, who refer to themselves as ‘Adivasis’, an umbrella term for several distinct communities (first citizen in many Indian languages). The Soliga, like other Adivasi communities, have been socially, politically and historically marginalized both during the colonial period and in post-independence India (Xaxa, 2016). They have lived in the Biligiriranga Hills and surrounding forested regions of Southern Karnataka for centuries but are relatively isolated from other communities that have remained outside of the forests (Morlote *et al.*, 2011). Despite decades of public health interventions and health service strengthening, the Soliga people’s health status remains poor (Xaxa, 2016; Srinivas *et al.*, 2019; Thresia *et al.*, 2020). Coverage and utilization of facilities for institutional deliveries and safe motherhood components in areas with indigenous populations is inadequate in many areas of India (George *et al.*, 2020). Even where services are available, their quality and timely utilization by indigenous women remains low (George *et al.*, 2020). In Karnataka, as per the most recent district survey (2007–08) at the time, the coverage of full antenatal checkups among women belonging to Scheduled Tribes (of which the Soliga is one) was 39.9% relative to 52.2% for others. Similarly, the difference of institutional childbirth coverage was 49.5% for tribal women as compared to 66.7% for others (International Institute for Population Sciences 2010). As such, it seemed important to design an intervention to improve Soliga women’s utilization of maternal and child health services.

Stage 1 of the priority-setting process consisted of developing a list of problems related to accessing maternal and child health services by collecting data from Soliga households in Chamarajanagar district and thematically analysing them. Stage 2 entailed prioritizing amongst the problems and identifying interventions through a deliberative workshop. Stage 3 consisted of selecting interventions to implement and this was achieved during a meeting amongst leaders of the Zilla Budakattu Girijana Abhivrudhhi Sangha (district Sangha) and researchers. Data on the priority-setting process were collected in 2018 and 2019 through in-depth interviews with researchers, Sangha leaders and field investigators from the Soliga community who collected the data on problems related to accessing maternal and child health services in Stage 1. Direct observation and document collection were also performed. Data from all sources were thematically analysed.

**Figure 1.**
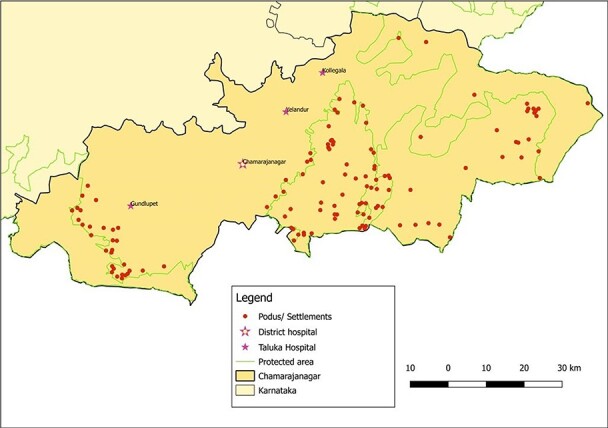
Distribution of hamlets of Soliga and other indigenous people in Chamarajanagar district

The paper first reports district Sangha characteristics in terms of its membership and decision-making processes and then describes who was represented and had voice across each phase of the priority-setting process. It demonstrates that, while CO-led priority-setting may share power and meaningfully include CO members in priority-setting, it comes with complexities to navigate. When COs lead research priority-setting, their strengths and weaknesses in terms of representation and voice will affect inclusivity at each stage of the process. Whether and to what extent CO partners’ decision-making processes are diverse and participatory affects who is represented/present and their numbers as well as who is able to raise their voice and be heard in priority-setting. Problematic gender dynamics within COs may be recreated, which is especially concerning when priority-setting focuses on improving women’s health. Based on these findings, recommendations are made to support the achievement of inclusive CO-led health research priority-setting processes.

## Methods

A case study of the Sangha-led priority-setting process was performed as part of a larger research project that examined sharing power with marginalized communities in health research priority-setting. Cases of priority-setting processes were selected where marginalized communities were meaningfully engaged in choosing research projects’ topics and questions and in designing interventions. The Sangha-led process was one such case.

Data were collected using a triangulation approach consisting of in-depth interviews (December 2018 and April 2019), document analysis and direct observation during a meeting between the researchers and district Sangha leaders (April 2019). Fourteen semi-structured in-depth interviews were conducted with researchers (four interviews), Sangha leaders (five interviews) and field investigators from the Soliga community (five interviews). Specific Sangha leaders and field investigators were suggested for interview by the district Sangha leader who supervised the field investigators. Efforts were made to select Sangha leaders and field investigators across genders and all four sub-districts within Chamarajanagar district (Figure 1), and field investigators with and without Sangha affiliation, but this proved difficult. Most field investigators who were interviewed were affiliated with the Sangha as either leaders or volunteers. In total, four women and eleven men were interviewed. To some extent, this reflected the fact that there were fewer female researchers than male and far fewer female Sangha leaders than male. Of the ten Sangha leader and field investigator interviewees, six were from Yelandur sub-district, 3 from Kollegal sub-district and one from Chamarajanagar sub-district. Seven were Sangha leaders and one volunteered with the Sangha. Two interviewees were not part of the Sangha organization.

Written informed consent was obtained from all participants. Following the technique of thick description, interview questions were open-ended (Geertz, 1973), and a subset of the questions attempted to draw out interviewees’ experience with and perspectives on inclusion during the priority-setting process (see Box 2).

**Box 2.** Interview guide

Interviewees were asked the following questions: What was important to achieving shared decision-making during the priority-setting process? How did the priority-setting process access the diverse voices and knowledge of community members, including those considered disadvantaged or marginalized?^2^ How did you decide which voices from the consultations to amplify and take into the deliberative workshop? Who was responsible for synthesizing the data from consultations and why? How were power imbalances minimized during the deliberative workshop? Whose voices and knowledge were present in the problems that were prioritized and the interventions developed to address them? The question guide was translated to Kannada and then back-translated to English to ensure questions retained their original meaning.

All interviews were conducted in person, thirteen by BP and one by NSN. Interviews with researchers were performed in English. Interviews with Sangha leaders and field investigators were performed in Kannada with the assistance of a research assistant (NSN). Interviews’ duration was 63–126 minutes.

A meeting between the district Sangha leaders and researchers was observed by BP and NSN in April 2019 to supplement the interview data. This meeting was not about the priority-setting process but a subsequent health systems research project being conducted by the Institute of Public Health and Vivekananda Girijana Kalyana Kendra researchers and district Sangha. Given the case study was retrospective, observing the meeting was considered the best option to observe the dynamics between the researchers and the Sangha. While the project of focus was different, the actors and setting involved were not. Observations during the meeting focused on relational and deliberative dynamics rather than meeting content. BP and NSN each took shorthand notes regarding who led the meeting, where people physically sat, who spoke, who was silent or listening, whether Sangha leaders challenged or disagreed with things the researchers said, who made decisions, whose voices were reflected in the decisions that were made, and whether consensus was reached. After the meeting, the two observers debriefed about what they had seen. As NSN spoke Kannada and BP did not, NSN’s observations largely held sway where there was a discrepancy. Priority-setting process-related documents were also collected, including the priority-setting process final report; videos about the Soliga people, their history and culture; and a film made about the collaboration between the researchers and Sangha leaders.

Interviews were transcribed verbatim and, where necessary, translated from Kannada to English. Thematic analysis of interview transcripts, notes from direct observation and debrief, the priority-setting process final report, and notes from videos was undertaken by two coders in the following five phases: initial coding framework creation, coding, inter-coder reliability and agreement assessment, coding framework modification, and final coding of the entire data set (Hruschka *et al.*, 2004; Campbell *et al.*, 2013).

### Authors’ positionality

The paper’s first author is an ethics researcher from a high-income country. She was not a member of the research partnership or part of the priority-setting process. This case study was her first introduction to the Sangha and its processes and the Soliga people, their history, and their way of life. She does not speak Kannada.

The remaining two authors were researchers who partnered with the Sangha to conduct the priority-setting process. Both are medical doctors and have worked in primary care at Vivekananda Girijana Kalyana Kendra hospital. In 2009, they set up a long-term tribal health research field station embedded within the Soliga community and with linkages to local health departments, NGOs and indigenous COs. They have purposefully limited their participation in designing and conducting the case study. They provided input on the study’s recruitment scripts, Plain Language Statement, Consent Form and Question Guide to ensure that they were appropriate and would be understood by study participants. After data were thematically analysed and an outline of this paper was written, they provided feedback. They then provided comments on a first draft of the paper. They also summarized the draft to the Sangha leadership, documented their responses and shared those responses with the lead author.^3^

## Results

First, the nature of representation and voice within the CO are discussed. Second, representation and voice are considered at each stage of the priority-setting process: (1) documentation, (2) analysis, (3) setting priorities and identifying interventions and (4) selecting an intervention. Where data presented came from observations or documents rather than interviews, this is explicitly mentioned.

### Features of the community organization

#### Representation

How representative are district Sangha leaders of the Soliga community and its diversity, including on demographics relevant to maternal and child health like gender? Sangha leaders are not elected. They change over every three  years and geographical location is considered when selecting leaders along with several other traits. The district Sangha is comprised of twenty-one members (district Sangha leaders), with four to five from each sub-district except Kollegal, which is larger and has seven representatives. The district Sangha leaders thus reflect the district’s geographic diversity. (At the sub-district level, Sangha leaders are selected from different village clusters to ensure that they are not all from the same villages or villages close to one another. Each village in the four sub-districts has its own Sangha as well.)

Amongst the district Sangha leaders, there are fewer female, young and relatively poor leaders, especially at the district and sub-district levels. Many of the district Sangha leaders are 40–50 years of age. Only five members of the district Sangha are women, but representation between the genders is more equal at village level. A female Sangha leader attributed this to women having a family to look after and being unable to leave their homes to travel to district and sub-district Sangha meetings. She noted that widows and women with children who have finished schooling and who have a desire to do social service and help others are more likely to be able to be district Sangha leaders, particularly if they are provided with a small allowance for their travel. Relatively impoverished individuals are not selected to become leaders because they are dependent on daily wages and thus typically do not have the time or resources to fulfil the duties of being a district Sangha leader (i.e. to travel to and attend meetings). Sangha leaders are all unpaid and carry out their role as volunteers. The district Sangha does not have the funds to provide leaders with a regular travel allowance to attend meetings.

The district Sangha is aware of its shortcomings in terms of youth and gender and is trying to address those gaps. However, its leaders emphasized the stratification of activities between district-, sub-district- and village-level Sanghas means that, by design, the district Sangha does not need to be highly representative, whereas sub-district and village Sanghas do. The district Sangha sees itself as a pressure group, working on policy issues and serving the interests of all Soliga in the district.

#### Voice

How participatory are district Sangha decision-making processes? Researchers and district Sangha leaders described district Sangha meetings as characterized by ‘deliberative norms’—having an equal opportunity to speak, turn-taking and listening, asking questions and receiving answers, dissenting, and reaching consensus. Even a single dissenting voice must be deliberated. A researcher (R03) thought such practice comprises ‘a respectful way of ensuring that every voice is heard even if others don’t agree with it.’ The Sangha also deliberate until consensus is reached, which, for them, means a majority (90% according to one interviewee) agree with a decision. If consensus cannot be reached, matters are tabled until their next meeting. Even where consensus is reached by a majority, things are not set in stone. Decisions can be revisited at subsequent meetings.

Both male and female leaders affirmed that everyone has an equal chance to speak at district Sangha meetings but acknowledged some speak more than others. According to a female interviewee,

*Some who have the knowledge about this, and some who are educated, and some who have information about this through other sources, they speak more about this, they asked lot of questions. People like us, with little knowledge, spoke less* (SL01).

She went on to say that men have more knowledge about what is going on in the Soliga community because they can go anywhere at any time, so they are able to interact more with people in different villages. Women, on the other hand, have responsibilities that keep them close to their homes. At the observed meeting in 2019, female Sangha leaders were present but rarely spoke and, when they did speak, it was primarily to agree with what male leaders had said rather than to voice ideas of their own.

Although it is mainly district and sub-district Sangha leaders who attend district Sangha meetings, the voices of the wider Soliga community inform their decision-making. Within the Sangha, communication flows from the village to the sub-district to the district and in the other direction. Problems arising at the village level reach the sub-district Sangha followed by the district Sangha. Similarly, issues discussed at the district level meetings are next discussed at sub-district meetings and then village meetings.^4^

Sangha leaders affirmed that this structure and increasing mobile phone use enables the district Sangha to access the voices of more remote and/or relatively poor members of the Soliga community. These more marginalized members of the community won’t come to district Sangha meetings. They raise their concerns and opinions at village-level meetings, and those issues are then discussed at sub-district- and district-level meetings. An interviewee noted, ‘when they discuss in village, there is no parity like rich or poor, everybody will attend the meeting, there will be female, male, young ones, and they participate in the discussion’ (SL02).

However, another Sangha leader suggested that information about problems from certain villages may not reach the district Sangha. Some village Sangha leaders do not attend higher-level Sangha meetings. The district leaders further acknowledge that being a volunteer organization means that voices from the most faraway villages can be marginalized and are not always heard by the district Sangha.

### Features of the priority-setting process

#### Developing a list of maternal and child health service access problems: step 1 documentation

##### Representation

Field investigators collected information on what maternal and child health service access problems were being experienced from all Soliga households in the four sub-districts in Chamarajanagar district. They and the priority-setting process final report reported collecting information from a range of people: village leaders (Sangha leaders, women’s collective organization leaders, and local government leaders); pregnant women; women with small children; health workers (local female traditional doctors, nurses, and frontline workers running tribal health mobile units); and heads of households, husbands, parents and other family members, including anyone else in households who was experiencing an illness.

More pregnant women and women with children were spoken to than village leaders and men, as men were often at work when field investigators came to their homes. Of these women, however, fewer women who had recently accessed health services were interviewed than would have been ideal, according to the researchers. Women who had accessed health services were more likely to have enough recent experience to give useful input.

Field investigators reported reaching relatively poor households, but the numbers varied from four to five such households, to twenty to twenty-five out of 250 households visited, to 100 of 200. They also reported that there was little if any marginalization or stigmatization of groups within the Soliga community when interviewers asked if they had collected information from marginalized groups. Two field investigators said that the only example they could think of was individuals who married men/women who were not Soliga. They said they had not come across such individuals during data collection.^5^

##### Voice

According to the field investigators, more information was collected from pregnant women and women with small children than village leaders. This reflected their getting detailed information from the women, whereas the leaders ‘were telling only about the population, they were not giving correct information’ (FI02).

#### Developing a list of maternal and child health service access problems: step 2 analysis

##### Representation

Once the data were collected, the priority-setting process final report states it was thematically analysed, generating a list of six key maternal and child health service access problems faced by the Soliga community. Field investigators and Sangha leaders identified three male district Sangha leaders from Yelandur as responsible for analysing the data. They said these leaders were selected because they were the only Sangha leaders who had the necessary analysis and writing skills. However, a researcher described it as a joint data analysis carried out by two researchers (a male and a female) and a male Sangha leader from Yelandur, supplemented by a joint data analysis workshop with the ten field investigators and regular brainstorms with the other district Sangha leaders.

##### Voice

When asked whose voices were reflected in the six themes or problems to accessing maternal and child health services, Sangha leaders reported that the problems encompassed all sub-districts and gave importance to local problems that affect the women first. A Sangha leader highlighted a distinction between three levels of people within the Soliga community and said that the six themes focused on the problems experienced by the third category—lower status people:

*People who are financially comfortable are getting everything, he is intelligent and aware of everything. He can get health benefits or any other benefits himself. Middle level person follow us. Lower level person doesn’t have money and he is not aware of whom to approach. Lower status people ask, we are in difficulty please help. Middle status say, ‘we know the hospital we talk to ANMs [auxiliary nurse midwives] and go’. People in top level say, ‘we go to Mysore I don’t require any of these things’… So we have to focus more on lower status people. We have to uplift them* (FI03).

Several field investigators had not seen the final report and thus could not comment.

Researchers noted that male Sangha leaders’ voices were also reflected in the six themes. At quarterly data analysis meetings, the district Sangha leaders would identify the importance or urgency of the problems reported in the data, at times disputing the village-level data. According to a researcher,

*they’d say oh but this isn’t the village voice, this is probably one person saying it, so there was also some sort of discussions like that where they would weigh in and say this isn’t important or this is important* (R03).

The weightage given to certain issues would be refined accordingly.

#### Setting priorities and identifying interventions: a deliberative workshop

A deliberative workshop was held that aimed to prioritize amongst the identified problems to accessing maternal and child health services and to devise a list of interventions to address them. As described in interviews and the priority-setting process final report, the workshop was structured as follows on Day 1: summary of the data collection and findings and small group work by sub-district, where each group identified its top three to four barriers to accessing maternal and child health services. The choice for small groups to be divided by sub-district came from workshop participants, not the researchers. On Day 2, the small groups reported back to the wider group, district Sangha leaders gave their comments, and the whole group ‘negotiated’ the collective list down to roughly ten priorities until consensus was reached. A whole group discussion then followed on what interventions or actions could be taken to address the prioritized problems.

##### Representation

District Sangha leaders and researchers reported the following types of people as present at the deliberative workshop: themselves, sub-district level Sangha leaders, village-level Sangha leaders, women’s organization leaders, villagers, youth leaders, field investigators, and research assistants from Vivekananda Girijana Kalyana Kendra and the Institute of Public Health. Women’s collective leaders are women who direct organizations for the economic empowerment of Soliga women. They were also not part of the Sangha. Villagers were from the Soliga community and had come to get help with accessing government health insurance schemes, so their presence was not exclusively for the workshop. Villagers included poor families from both close by and far away. Their attendance was facilitated by Sangha leaders either covering their transport costs or bringing them to the workshop in their cars.

The workshop was attended by fewer women than men. Three women—all from Yelandur—and 57 men attended. An interviewee further noted a lack of elderly women and young unmarried women being present. There were also fewer participants from Gundlupet and Chamarajanagar sub-districts relative to Yelandur and Kollegal sub-districts. This may have been a reflection on who the district Sangha invited to the workshop, with one leader affirming that the organization invited who they always invite: those who live nearby and can travel to the venue in a relatively short amount of time.

##### Voice

According to researchers and Sangha leaders, discussions in the small and large groups were characterized by the deliberative norms that are a feature of Sangha meetings: ‘We give open chance to anybody to give their ideas, we do not snub them, whatever their problem, we give them a chance to discuss it openly’ (SL03). Participants also listened to one another, which ensured that problems experienced in different sub-districts were considered. Voices of dissent were heard and discussed, and ultimately consensus was reached, and everyone agreed to the final list of priorities.

Small group work was undertaken to help everyone feel comfortable raising their voices. This was largely successful, with several Sangha leaders (SL03, FI05) noting that ‘all with opinions shared them’, that it was a very ‘open discussion’ and that ‘everyone talked in the small groups’, including women and villagers. Since field investigators had visited households as part of data collection, everyone in the villages knew about the priority-setting process and villagers were thus able to contribute in the small group discussions. These discussions also purposefully served to minimize district Sangha leaders’ voices. They acted as commentators once the groups reported back, rather than as participants in the small group work.

However, in the wider group discussion that generated the final list of priorities, there was more participation by male district Sangha leaders from Yelandur compared to women and villagers. Although the female leaders’ perspectives were raised in the Yelandur sub-district small group, their lack of numbers meant that their voices were heard less in the final selection of priorities. According to one researcher, villagers did not speak at all during the discussion. The final prioritization was based on a male leader perspective.

In deciding what problem to intervene on and in devising a list of interventions, researchers felt that their voices and male Sangha leaders at state and district levels were raised and heard much more than other participants. At the deliberative workshop, women leaders called for implementing an intervention to target alcoholism. Yet their recommendation could not be taken forward because existing funding focused on maternal and child health service delivery. Given the research team’s primary intentions of improving health services and systems in turn linked to the funder’s primary intention of focusing on maternal and child health, the team tended towards interventions that were identifiably located within health services and systems. The issue of widespread alcohol use brought up by the women were seen (within the research team) as being further away from health services and systems. The researchers reported having to drop hints at the workshop that such interventions would have to be funded and taken up outside of the current priority-setting process space.^6^ They described this as ‘exercising our power’ to ensure that ‘activities that are more amenable to being an intervention in our lens, like, having a [helpline] for example they took more priority’ (R04).

The male district leaders’ remarks also ‘shifted the dynamic’ by pushing for certain interventions as possible solutions to the problems on the list. The helpline intervention that was ultimately implemented was first proposed by a state-level male Sangha leader. The absence of women thus affected the types of interventions proposed to address health problems that significantly impact them. A researcher affirmed, women likely would have come up with a different intervention than the helpline:

*I find that they, they see a problem and the way they feel it should be addressed is very different from the male leaders here, because for them things are political, protest, petition. For the women leaders it’s more hands-on, what can we do, call the doctor, do this, do that so more awareness education* (R03).

#### Selecting an intervention

After the workshop, the researchers and several male district Sangha leaders made a final decision on which intervention(s) they would implement with existing resources. This decision was then discussed and approved at a district Sangha meeting. It is unclear if sub-strict and village Sanghas also discussed and approved the intervention.

## Discussion

Funders, research institutions and ethicists increasingly push for community engagement in health research. One way thought to achieve meaningful inclusion for communities throughout health research projects, including during priority-setting, is for researchers to partner with COs. Yet, if community voice is included without a closer examination of the internal power dynamics within the CO partner, then inclusion may simply exacerbate or reinforce the very hierarchies that impede the achievement of improved health outcomes, e.g. exclusion of women in decision-making processes related to their health and their bodies. These lessons have been clearly shown in participatory development. As Andrea Cornwall (2001, p. 1, 15) affirms, ‘inviting the “community” to design their own intervention runs the risk, however, of reinforcing other stakes: those that maintain the status quo… The very projects that appear so apparently transformative in terms of “local people” exercising voice, choice, managing and solving problems for themselves, can turn out on closer inspection to be supportive of a status quo that is highly inequitable for women’.

This paper provides initial empirical evidence as to the complexity of partnering with COs in research priority-setting, in the context of a pre-dominantly non-indigenous research community from the ‘outside’ partnering with the leadership of an indigenous CO. Below, we consider our findings in the light of the different components of representation and voice described in the broader literature on participation: range, mass, raising voice, and being heard (see Young, 2000; Goodin, 2008; Crocker, 2008; Cornwall, 2011; Pratt, 2018). We show that CO strengths and weaknesses relate to and affect the different components of representation and voice at each stage of the priority-setting process: (1) developing a list of health problems or possible research topics, (2) prioritizing amongst the health problems and (3) selecting research questions and interventions. CO weaknesses in terms of female representation are particularly worrisome in the case under study due to its being a maternal and child health priority-setting process.

CO strengths can deepen inclusion by the CO and its wider community in health research priority-setting; CO weaknesses can create limitations for inclusion if not mitigated. The district Sangha’s strengths were having a robust representation of geographical diversity amongst its leaders and members and deliberative decision-making processes that gave attendees an equal opportunity to speak at meetings. The former strength contributes to achieving range and the latter to raising voice. Robust representation meant that data on problems related to accessing maternal and child health services were collected from all households in the district, including the relatively poor. That data then captured the voices of women at upper, middle, and lower levels of Soliga society, with analysis by Sangha leaders ensuring that the experiences of women at the lower level were strongly reflected in identified themes. At the deliberative workshop, Sangha leaders from all four sub-districts participated. Some villagers were also present, including the relatively poor. The Sangha’s geographical diversity thus promoted having a range of participants. Since norms of deliberation are a feature of Sangha meetings and decision-making, the deliberative workshop largely operated according to those norms, which promoted (but did not ensure) participants raising their voices and being heard.

However, the district Sangha’s own gender imbalances impeded greater female participation in the latter two stages of the priority-setting process. While data were collected from a diversity of women, no female leaders participated in the analysis or in the final selection of the intervention and few were present at the deliberative workshop. In part, their lack of numbers was due to the district Sangha having many fewer female leaders than male leaders. Reasons given for women’s lack of participation in the district Sangha (i.e. time constraints and location being incompatible with their work roles) are consistent with those in the gender development literature (Agarwal, 1997; Cornwall, 2001). A parallel process for women was not run due to the researchers’ concerns about seeding a new short-term engagement process to primarily serve their research agenda. From an inclusion perspective, this impeded achieving a range of participants and a sufficient mass of female participants in priority-setting. At times, power dynamics related to knowledge also deterred some female leaders from speaking up in relation to community health matters. Men raised their voices more than women, which in turn led to disparities in who was heard during priority-setting. Women’s perspectives were not strongly reflected in the identified priorities or interventions.

This state of affairs differs somewhat from existing evidence that shows being female means that community members are listened to less or not at all in health priority-setting (Shayo *et al.*, 2012). In the Sangha case, the issue was more a lack of female presence and lack of female confidence raising their voices about community health matters, rather than an example of men failing to listen to what women said. The researchers did, however, purposefully ignore female Sangha leaders’ comments about alcoholism in relation to the particular priority-setting process serving as our case.

In the context of a maternal and child health priority-setting process, the lack of female presence and voice is especially troubling. It means the continued exclusion of women in decision-making processes related to their health and their bodies. Concerns about women’s inclusion have been strongly raised in participatory development (Agarwal, 1997; Cornwall, 2001). Their participation and representation in decision-making structures is identified as a key aspect of women’s empowerment (Kabeer, 2005). Exclusion of women adversely affects their power to participate in decisions that affect them, to advocate for their interests, and to push back against decisions made by others that may affect women. The importance of their inclusion has been shown. As Kabeer (2005, p. 22) reports, studies from India demonstrate women ‘questioned the priorities of *panchayat* (local government) development programmes, emphasized issues affecting women such as fuel and water, and had begun to build broad alliances among themselves. One study showed that women representatives were likely to allocate resources differently from men, suggesting that their presence allowed a different set of priorities to be expressed’. Similarly, in this case study, women raised different priorities (alcoholism) and were thought likely to prioritize their health and health system needs differently to men.

Women’s lack of inclusion was acknowledged by Sangha leaders and discussed many times throughout the course of the priority-setting process. So far, barriers to women’s participation in the district Sangha have been identified, but efforts to address them have not been hugely effective. Evidence about strategies for deepening women’s participation from development studies may offer useful guidance for them going forward (Cornwall, 2001; Mangubhai and Capraro 2015).

Another issue was that priority-setting achieved different levels of participation for the CO and the wider Soliga community. Sherry Arnstein (1969), Crocker (2008) and many others have distinguished amongst different levels of participation. Collaboration or partnership involves shared decision-making between parties. Consultation is characterized by one party being invited to give their input but having no assurance that it will be used by those who decide. Deep inclusion has been defined as both involving a diversity of community members, including those considered disadvantaged or marginalized, and involving them as decision-makers (Young, 2000; Oswald *et al.*, 2016). In the case under study, the wider community participated as a consultant, while the Sangha participated as a partner. This had implications for range, mass, and voice during the process.

Despite the Sangha’s strong links with the wider community through its sub-district and village-level organizations, the wider community did not participate in data analysis or in the final selection of the intervention. Fewer members of the wider community were present at the deliberative workshop relative to Sangha leaders and did not raise their voices in the full group discussion. As previously noted, villagers, including the relatively poor, typically attend village Sangha meetings rather than district Sangha meetings. They feel comfortable speaking up at village-level Sangha meetings but less frequently attend district-level gatherings. As such, they may not have felt confident expressing themselves at the deliberative workshop. The wider community’s lack of presence and voice meant that decision-making on what barriers to accessing maternal and child health services were prioritized and what interventions should address them came primarily from the Sangha leaders and researchers. While it is true that some Sangha leaders came from the village Sanghas, not hearing from Soliga women, in particular, when prioritizing amongst the maternal and child health problems and selecting interventions was problematic. It again meant that women were excluded from decision-making about matters related to their health. In future priority-setting exercises led by the district Sangha, strategies to mitigate low community representation, especially of women, and to draw out their voices during deliberations should be enacted.

Finally, the study highlights how the layered and intersectional nature of identity, which has been previously well documented (Mangubhai and Capraro 2015; Larson *et al.*, 2016), can affect participation. Axes of powerlessness are intersecting and context-specific (Cornwall, 2008). In India, indigeneity and gender (amongst other characteristics) are associated with marginalization and exclusion. Attempts by the researchers to include one axis of powerlessness (indigeneity) in this case study ended up reinforcing another axis of powerlessness (gender). It is also essential to recognize that women experience many different identities simultaneously and that there are likely to be divisions amongst Indian women based upon, for example, marital and family status (Mangubhai and Capraro 2015). It is important that less heard and less powerful women in the Soliga community are involved in future priority-setting exercises. As previously noted, the deliberative workshop was not simply lacking participation by female Sangha leaders and women from the community; it lacked participation by elderly and unmarried women.

**Box 3.** Recommendations for researchers and community partners

We recommend researchers and community partners reflect on CO partners’ strengths and weaknesses in terms of representation and voice before starting a CO-led priority-setting process. During these reflections, it is important to return to the different components of representation and voice: range, mass, raising voice and being heard. This means identifying specific CO strengths and weakness; assessing how they affect range, mass, raising voice, and being heard during each stage of priority-setting; and devising strategies for mitigating the effects of any weaknesses on the priority-setting process. When doing so, using an intersectional lens informed by axes of powerlessness in a given community will be important. Wherever possible, CO assets should be utilized in mitigation strategies. Supplementary File 1 provides researchers and their community partners with a guide to use to undertake such reflections. It offers considerations to help enhance inclusivity in CO-led priority-setting processes by building on CO strengths and addressing CO weaknesses. In this way, our recommended approach is both necessarily asset and deficit-based.^7^ The guide reflects the priority-setting process used in the case described in this paper, but it can be adapted for use in processes that employ a different mix of consultative and/or deliberative stages.

In light of our findings, we offer recommendations for researchers and community partners in Box 3. These recommendations are intended to help them achieve more inclusive priority-setting processes. We also note that the case study had several limitations. Members of the Soliga community who were consulted as part of the data collection phase were not able to be interviewed because records of their identities were not kept. Ideally, more women, more Sangha leaders from Chamarajanagar and Gundlupet sub-districts, and more non-Sangha field investigators would have been interviewed as well. The latter were sought to offer more impartial perspectives on whether the CO was a good representative of the Soliga community. Efforts were made to interview individuals in these categories, but finding available interviewees proved difficult.

Given the case study was carried out more than four years after the priority-setting process occurred, several interviewees had poor recall of what happened during the process. Where this happened, it was noted in the field notes for the particular interview. It was then taken into account when coding. Parts of interviews were excluded where an interviewee did not remember an event or were coded under the category ‘general Sangha processes’, where an interviewee described how things generally happened in Sangha meetings rather than what had happened in the priority-setting process meetings with researchers. The retrospective nature of the case study also meant that meetings between the researchers and Sangha leaders and the deliberative workshop were not directly observed. To supplement interview data, a 2019 meeting between Sangha leaders and the Institute of Public Health and Vivekananda Girijana Kalyana Kendra researchers was observed.

Future research could usefully explore how CO characteristics in terms of representation and voice affect priority-setting in other types of health research (e.g. not health systems research) and in pure deliberative or consultative processes. It could also be conducted during priority-setting processes (as opposed to afterwards) in order to gather robust direct observation data from consultations and deliberations in terms of the range and mass of participants, who speaks, and who is heard. Future research could explore how community inclusion, or the necessity of such inclusion, affects the research organization partner. It could investigate whether reflecting on the considerations in Supplementary File 1 lead the district Sangha (or other COs) to set up more inclusive CO-led priority-setting than it or they had previously. The district Sangha continues to collaborate with the Institute of Public Health and Vivekananda Girijana Kalyana Kendra researchers on new projects.

Ultimately, despite the case study’s limitations, we hope that the strategies and learnings presented in this paper help facilitate more inclusive CO-led health research agenda setting as part of CO–researcher partnerships. Inclusion in research decision-making of those who have traditionally been excluded can bring different, previously underrepresented perspectives into research and help redress power imbalances in the production of knowledge. But to do so it requires being aware of CO strengths and weaknesses and navigating the resultant complexities during engagement.

## Supplementary Material

czac012_Supp

## Data Availability

The data underlying this article will be shared on reasonable request to the corresponding author.

## Endnotes

1. Since 2017, the participatory research that began with maternal health has evolved into an initiative to understand, explain and act on the drivers of health inequities experienced by the Soliga through community-based and health system interventions.

2. The idea of entire communities and castes being disadvantaged through social (structural and institutional) factors has widespread penetration within social movements of the Dalit and Adivasi communities in India. These movements often have local words for concepts used in the development literature like disadvantage, oppression or marginalization. Thanks to these social movements, Kannada equivalents of those local words exist and have a use amongst the Soliga community.

3. Based on Sangha leaders’ comments, minor changes were made to the features of the district Sangha reported in the paper. The leaders’ following comments were incorporated: (1) that, by design, the district Sangha does not need to be highly representative and (2) that being a volunteer organization means that voices from the most faraway villages can be marginalized and are not always heard by the district Sangha.

4. In such cases, district Sangha decisions may be in tension with sub-district Sangha decisions. Continued discussions between Sangha organizations allow for eventual consensus and convergence in decision-making.

5. This reflects the typical practice of such couples leaving Soliga villages to live elsewhere.

6. Since then, the research team has worked alcohol use—both its academic research element and participatory program building element—into a future research grant that finally materialized in October 2021. This programme has now begun and hopes to work closely with women’s groups in the coming years to provide de-addiction services and build wellness activities among youth to prevent early addictions among tribal youth.

7. The premise of asset-based approaches is that communities can drive the development process themselves by identifying and mobilizing existing (but often unrecognized) assets, whereas deficit-based approaches focus on ‘experts’ (from universities, donor agencies and governments) identifying what communities lack and developing solutions to address those needs (Nel, 2018).
