# Supplementary material for: How is inclusiveness in health systems research priority-setting affected when community organizations lead the process?
Source: Health Policy Plan. 2022 Feb 16;37(7):811–21. doi: 10.1093/heapol/czac012 (PMC9347025; doi:10.1093/heapol/czac012)
Supplement: czac012_Supp [file czac012_supp.zip › Supplementary File 1.docx]

**Supplementary File 1: Considerations to help enhance inclusivity in CO-led priority-setting processes**

1. Identify CO strengths and weaknesses in terms of range, mass, raising voice and being heard for its leaders and member of its wider community.

|  | **Range**: Diversity of leaders and members of the wider community reached or represented, including those considered disadvantaged, relatively poor, or marginalised | **Mass**:  Numbers of different types of leaders and community members CO reaches or represents | **Raising of voice**:  Sharing of ideas and views by the diversity of leaders and the wider community, including those considered disadvantaged, relatively poor, or marginalised | **Being heard:** Ideas and views of a range of leaders and community members are incorporated into CO decisions |
| --- | --- | --- | --- | --- |
| **Strengths** |  |  |  |  |
| **Weaknesses** |  |  |  |  |

2. Reflect upon the following considerations *before* starting a CO-led priority-setting process:

|  | **Range** | **Mass** | **Raising voice** | **Being heard** |
| --- | --- | --- | --- | --- |
| **Stage 1A- Data collection to identify research topics** | How do the CO’s strengths and weaknesses affect the range of people from whom data is being collected? | How do the CO’s strengths and weaknesses affect the numbers of different types of people from whom data is being collected? | How do the CO’s strengths and weaknesses affect who is likely or able to raise their voice during data collection? | NA |
| **Stage 1B- Data analysis to identify research topics** | How do the CO’s strengths and weaknesses affect the range of people who will be analysing data? | How do the CO’s strengths and weaknesses affect the numbers of different types of people who will be analysing data? | NA | How do the CO’s strengths and weaknesses affect whose voices are likely to be heard during data analysis? |
| **Stage 2- Deliberative workshop to identify research priorities** | How do the CO’s strengths and weaknesses affect the range of people who will be at the deliberative workshop? | How do the CO’s strengths and weaknesses affect the numbers of different types of people who will be at the deliberative workshop? | How do the CO’s strengths and weaknesses affect who is likely or able to raise their voice during the deliberative workshop? | How do the CO’s strengths and weaknesses affect who is likely to be heard during the deliberative workshop? |
| **Stage 3- Deliberations to identify research questions and interventions** | Same as Stage 2 | Same as Stage 2 | Same as Stage 2 | Same as Stage 2 |

3. Devise strategies to address the impacts of any CO weaknesses on the priority-setting process. Utilise CO assets wherever possible.

|  | **Strategies** |
| --- | --- |
| **Stage 1A- Data collection to identify research topics** |  |
| **Stage 1B- Data analysis to identify research topics** |  |
| **Stage 2- Deliberative workshop to identify research priorities** |  |
| **Stage 3- Deliberations to identify research questions and interventions** |  |
